# *In Situ* Focused Beam Reflectance Measurement (FBRM), Attenuated Total Reflectance Fourier Transform Infrared (ATR-FTIR) and Raman Characterization of the Polymorphic Transformation of Carbamazepine

**DOI:** 10.3390/pharmaceutics4010164

**Published:** 2012-02-09

**Authors:** Yingying Zhao, Ying Bao, Jingkang Wang, Sohrab Rohani

**Affiliations:** 1 State Key Laboratory of Chemical Engineering, Tianjin University, Tianjin 300072, China; Email: yzhao352@uwo.ca (Y.Z.); yingbao@tju.edu.cn (Y.B.); jkwang@tju.edu.cn (J.W.); 2 Department of Chemical and Biochemical Engineering, the University of Western Ontario, London, ON N6A 5B9, Canada; 3 Engineering Research Center of Seawater Utilization Technology of Ministry of Education, Hebei University of Technology, Tianjin 300130, China

**Keywords:** carbamazepine, polymorph transformation, *in-situ* quantitative method, *in-situ* analytical techniques

## Abstract

The objective of this work was to study the polymorphic transformation of carbamazepine from Form II to Form III in 1-propanol during seeded isothermal batch crystallization. First, the pure Form II and Form III were obtained and characterized. Then their solubilities and metastable zone limits were measured by *in-situ* attenuated total reflectance Fourier transform infrared (ATR-FTIR) spectroscopy and focused beam reflectance measurement (FBRM). A transition temperature at about 34.2 °C was deduced suggesting the enantiotropic nature of this compound over the studied temperature range. To quantify the polymorph ratio during the transformation process, a new *in-situ* quantitative method was developed to measure the fraction of Form II by Raman spectroscopy. Successful tracking of the nucleation of the stable form and the transformation from Form II to Form III during isothermal crystallization was achieved by Raman spectroscopy and FBRM. The results from these three *in-situ* techniques, FBRM, FTIR and Raman were consistent with each other. The results showed a strong dependency on the amount of seeds added during isothermal crystallization.

## 1. Introduction

Polymorphism refers to the ability of a molecule to have more than one crystal form in the solid state. Polymorphs have different physical and chemical properties, such as lattice energy, melting point, heat of fusion, solubility, dissolution rate, density and processability. Such differences may affect stability, formulation, potency, bioavailability and storage of pharmaceuticals and their performance [1]. As for intellectual property, a new polymorph can be patented if it shows better properties than a previously patented polymorph and a rival company may legally sell the same drug substance in a different crystal form [2]. Different polymorphs can be obtained by changing the crystallization conditions, e.g., the operating temperature, type of solvent, pH of the solution.

A successful industrial crystallization typically requires the development of a robust process in the laboratory including the knowledge of the solubility curve and the stability of the solution in the vicinity of the equilibrium point, as indicated by the metastable zone width. An understanding of the thermodynamics of the polymorphic systems and the mechanism of the phase transformation is a prerequisite for controlling the solid phase during the manufacturing of drug products. The polymorphic forms of a compound may exist as enantiomorphs or monomorphs [1]. If a substance has two forms, and one form is stable over a range of temperature and pressure levels, while the other form is unstable, the transformation will be reversible and the polymorphism is referred to as enantiotropic. When the transition temperature is below the melting point, the Gibbs free energy of the two solid forms is identical and the free energy curves intersect at this point at constant pressure. If one form is unstable under all conditions, the polymorphism is monotropic and the transformation is irreversible.

There are two proposed mechanisms for polymorphic transformation, *i.e.*, solid-solid transformation (SST) and solution-mediated transformation (SMT). The SST is thought to occur in the solid state phase from the metastable crystal structure rearranging into more stable structure without passing through the liquid or vapor phases [2]. During solid-solid transformation, the transition temperature is determined by observing the direction of movement of the interface between the two forms during the heating and cooling processes. In contrast, a solvent is involved during the SMT and the transformation is usually driven by the differences in solubilities of the two forms in the solvent. The mechanism of SMT typically involves three steps [3]. First, the stable phase in which the solution is less soluble, nucleates and grows; secondly, the solution dissolves in the metastable phase which makes the solution, in the third phase, reach and then exceed the solubility of the stable form. This is followed by the nucleation and growth of the stable form at the expense of dissolution of the metastable crystals. Therefore, the dissolution of the metastable crystals and the growth of stable nuclei compete and occur according to the relative kinetics of the dissolution of the metastable form and the crystallization of the stable form.

The study of the polymorphic transformation by *in-situ* technologies has been the focus of many investigations over the past few years. *In-situ* FBRM, PVM and ATR-FTIR were applied to monitor the polymorphic transformation of d-mannitol by O’Sullivan *et al.* [4,5]. Ono *et al.* [6] carried out the batch transformation experiments of l-glutamic acid at several temperatures with quantification of polymorphic fraction by Raman. The conversion kinetics of carbamazepine polymorphs to the dihydrate in aqueous suspension were studied using Raman spectroscopy by Tian *et al.* [7].

As Raman spectroscopy enables rapid, non-destructive measurements, the technique appears to be most promising for on-line process monitoring and analysis in the pharmaceutical industry [6,8,9]. However, there has been no effective quantitative method to calculate the polymorph ratio during real time crystallization by *in-situ* Raman.

Fourier transform infrared (FT-IR) and FBRM are important complementary tools for on-line solution concentration measurement and chord length distribution measurement of a solid phase. On some occasions, the FBRM has been reported to monitor the identification of morphology change during polymorphic transformation [5,10]. These spectroscopic investigations deliver chemical and physical information and combine high speed analysis and the non-invasive measurements with high selectivity and sensitivity.

Carbamazepine (CBZ) is used in the treatment of epilepsy and trigeminal neuralgia [11]. Four polymorphs and a hydrate as well as other solvates of CBZ have been reported in the literature [7,12,13]. Form III is most stable at room temperature and is used in the marketed tablets. However, Form II has been observed during processing and contributes to the decreased purity of CBZ Form III. Characterizing Form II and III and investigating their thermodynamic relationship are very important to enhance the purity of Form III. Therefore, the first objective of this work was to obtain pure CBZ metastable Form II and stable Form III, and then determine their relationship according to the thermodynamic laws developed by Burger and Ramberger [14]. 1-Propanol was chosen as the solvent and the metastable Form II was crystallized by rapid cooling of a highly supersaturated solution and then characterized by differential scanning calorimeter (DSC) and X-ray powder diffraction (XRPD). Then the solubility and metastable zone of the two forms were measured by *in-situ* ATR-FTIR and FBRM. A new quantitative method was applied to calculate the ratio between Form II and Form III in the real time monitoring polymorph transformation using *in-situ* Raman. The effects of seed loading in the isothermal crystallization on the polymorphic transformation were studied by combining the *in-situ* FBRM, FTIR and Raman.

## 2. Material and Methods

Carbamazepine was recrystallized by cooling crystallization. 1-Propanol was 99+% reagent grade, supplied by Alfa Aesar Johnson Matthey Company (Lancs, UK).

All crystallization experiments were performed in a 250 mL jacketed glass vessel. A Neslab RTE digital plus 740 bath circulator (Portmouth, NH) was used for temperature control. A Teflon-coated thermocouple was used for reading the temperature in the flask. For mixing, a top-mounted, two-bladed, flat electromagnetically driven stirrer was employed.

The X-ray powder diffraction (XRPD) spectra were obtained on a Rigaku-Miniflex powder diffractometer (Carlsbad, CA) using monochromatic radiation (30 kV and 15 mA) in the range of 2θ from 5.0° to 40.0°, at a step size of 0.05° with a counting time of 5 s for each step. The heat of fusion and melting behavior of solid samples were determined by a Mettler Toledo DSC 822e differential scanning calorimeter (Greifensee, Switzerland) with a heating rate of 10 °C/min. An ATR-FTIR (Hamilton Sundstrand, Pomona, CA, USA) was used for solubility measurements and solubility limits determination. A FBRM (Lasentec, Redmond, WA, USA) was used with a measurement duration of 10 s to detect the onset of crystallization and polymorphic transformation based on the number of the particles. The fraction of each form suspended in solution during the isothermal polymorphic transformation process was determined at 25 °C by a Raman spectrometer (Kaiser Optical Systems, Inc. RXN1-785).

### 2.1. Preparation of Pure Carbamazepine Polymorphs

In accordance with the Ostwald’s rule of stages, the cooling of polymorphic substances often first yields the least stable polymorph, which subsequently rearranges into the stable form [15]. It can be concluded from the related literature that both the cooling rate and the initial concentration influence the polymorphic distribution in a cooling crystallization from a single solvent [16,17]. According to McCrone [18], in a poor solvent the rate of transformation of a metastable form to a more stable polymorph is slower. Hence, a metastable form once crystallized can be harvested before it is converted to a more stable phase by solution mediated transformation. The solution (Form II in 1-propanol) was filtered approximately 5 min after the start of nucleation and dried overnight in a vacuum oven at room temperature. The dried Form II was relatively stable, and no transformation to Form III was observed by XRPD and DSC. Form III was recrystallized by cooling from 70 °C to 20 °C at a rate of 10 °C/h in anhydrous ethanol.

### 2.2. Polymorphic Transformation during Seeded Isothermal Crystallization

Initially 122.5 g 1-propanol was added to a 250 mL jacketed glass vessel and kept at 25 °C while collecting the FTIR and Raman reference spectra. Then 3.37 g carbamazepine was added to the solvent and the content was heated to obtain a clear solution with all the crystal counts shown by FBRM close to zero. The solution was cooled slowly to 25 °C, afterwards, maintained at this temperature for 1 h. Then crystals of Form II were added to the solution and the on-line monitoring by FBRM, FTIR and Raman was continued until the end of the experiment. At 25 °C, according to the later solubility measurement results, this solution was saturated with respect to Form II but supersaturated for Form III. Therefore, the polymorphic transformations from Form II to Form III without the nucleation and growth of Form II were studied.

## 3. Results and Discussion

The results are divided into four sections, characterization of carbamazepine polymorphs; determination of thermodynamic stability between Form II and Form III; quantitative analysis of polymorphic transformation; and real-time monitoring of polymorphic transformation of carbamazepine during isothermal crystallization. To avoid the impact of different experimental batches, all the seeds were from the same batch.

### 3.1. Characterization of Carbamazepine Polymorphs

**Figure 1:**
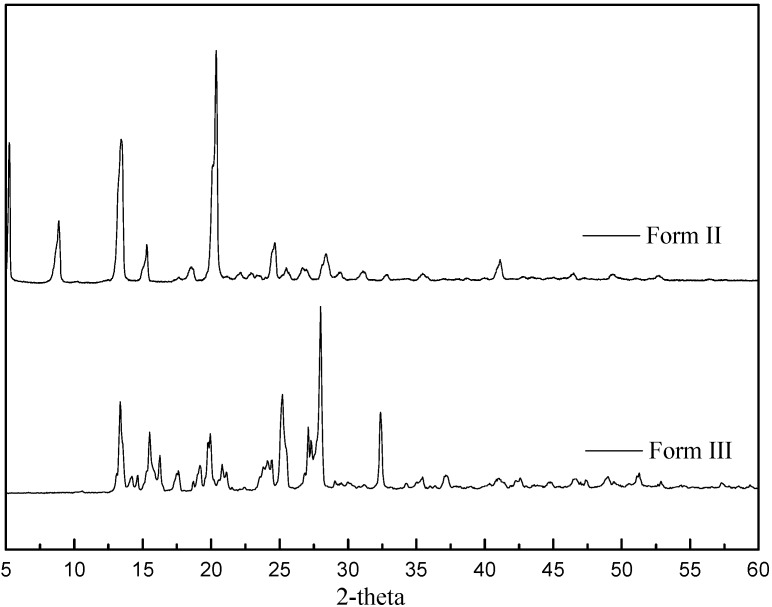
X-ray diffraction patterns of carbamazepine Form II and III.

The X-ray diffraction patterns of CBZ Forms II and III, shown in Figure 1, were found to be identical with the reported patterns in the literatures [7,12,13,19].

**Figure 2:**
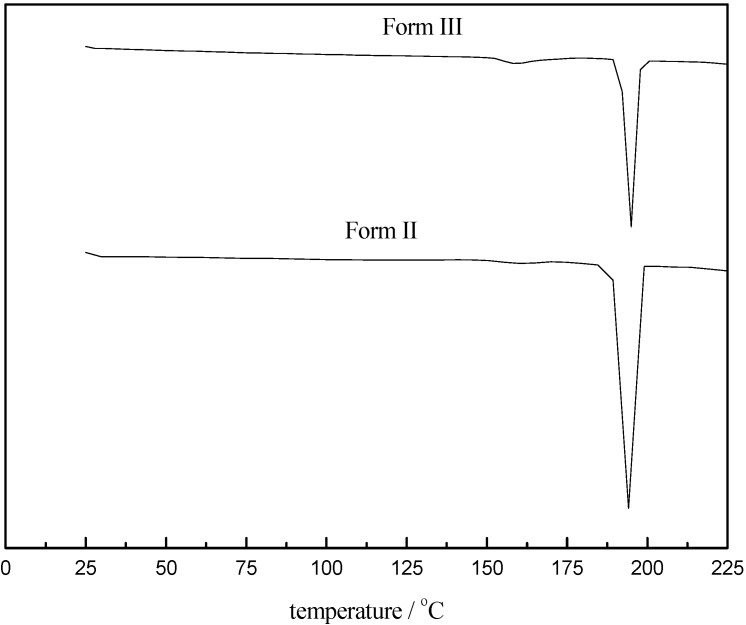
Differential scanning calorimetric curves of carbamazepine.

The heat of fusion and melting behavior of solid samples were determined by DSC at a heating rate 10 °C/min. Figure 2 shows the DSC curves of the prepared CBZ, which exhibit endothermic peaks at 189.8 °C and 191.4 °C for Form II and Form III respectively, which are within 2 °C of 188.1 °C, 189.2 °C reported by Yoshihashi *et al.* [13].

**Figure 3:**
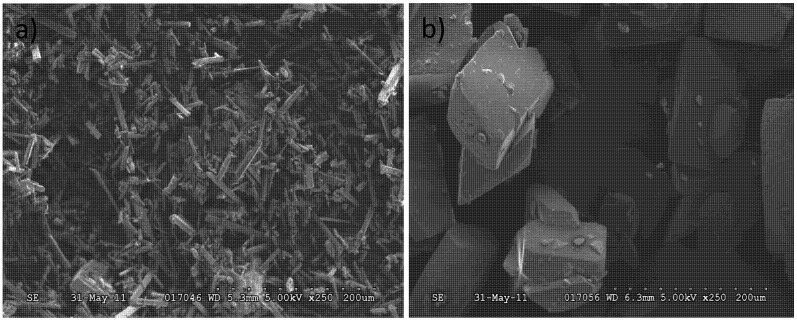
SEM pictures of polymorphic forms of carbamazepine: (a) Form II; (b) Form III.

Figure 3 depicts the SEM micrographs of Form II and Form III. The Form III crystals are plate shaped while Form II crystals are needle-like. Since the two forms have different crystal habits, their transformation can be detected by FBRM as well as Raman.

**Figure 4:**
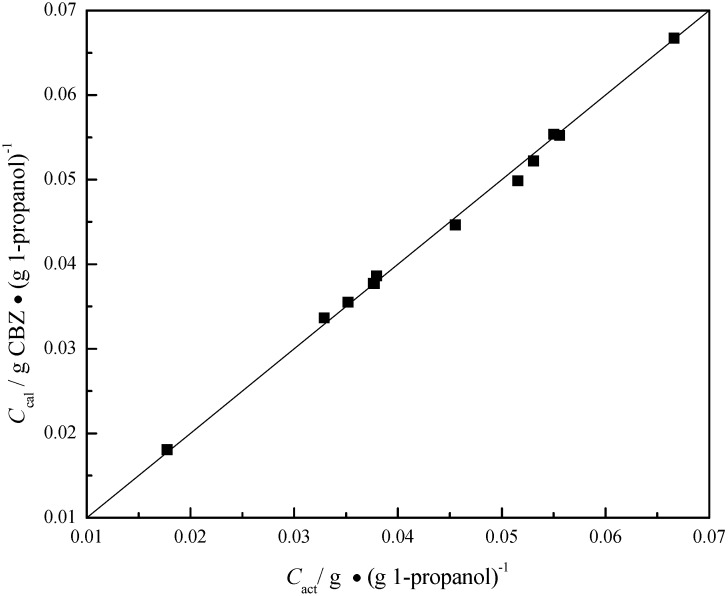
Comparison between actual and calculated concentrations.

The FBRM (Lasentec, Redmond, WA, USA) was used with a scan duration of 10 s to detect the appearance and disappearance of crystals based on the number of the particles in the chord length range of 1–20 µm during the solubility and metastable limits measurements. ATR-FTIR (Hamilton Sundstrand, Pomona, CA, USA) was used for the dissolved CBZ concentration measurement. The spectrum of 1-propanol at room temperature was used as the background for each sample. Mao *et al.* [20] have demonstrated that the relative peak proportionally increased with the solute concentration and was almost insensitive to temperature. In order to reduce the effect of noise, the absorbance intensity at 1251 cm^−1^ was subtracted from absorbance intensity peak heights at 1400 and 1677 cm^−1^. The calibration model is:

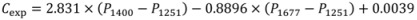
(1)
where *C*_exp_ is the CBZ concentration calculated from the ATR-FTIR measurements. *P*_1251_, *P*_1400_, and *P*_1677_ are the intensity values of absorbance peaks at 1251, 1400, and 1677 cm^−1^, respectively. To check the veracity of this model, some comparisons between the known concentrations and those calculated from the calibration model at different temperatures are shown in Figure 4 and Table 1, indicating that the concentration obtained from the calibration model can accurately represent the actual solute concentration to within a 95% measurement error of ±3.6%.

**Table 1:** Comparison between actual and calculated concentrations.

T/°C	*C*_act_/g ∙ (g 1-propanol)^−1^	*C*_cal_/g ∙ (g 1-propanol)^−1^	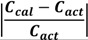
25.0	0.0177	0.0181	0.018
43.0	0.0329	0.0337	0.023
40.0	0.0352	0.0355	0.008
47.0	0.0376	0.0377	0.003
46.0	0.0377	0.0377	0.001
45.0	0.0379	0.0386	0.017
49.0	0.0455	0.0446	0.020
50.0	0.0515	0.0498	0.033
56.0	0.0531	0.0522	0.016
54.0	0.0550	0.0554	0.006
50.0	0.0556	0.0552	0.006
58.0	0.0666	0.0667	0.002

### 3.2. Solubility of Carbamazepine Polymorphs

**Figure 5:**
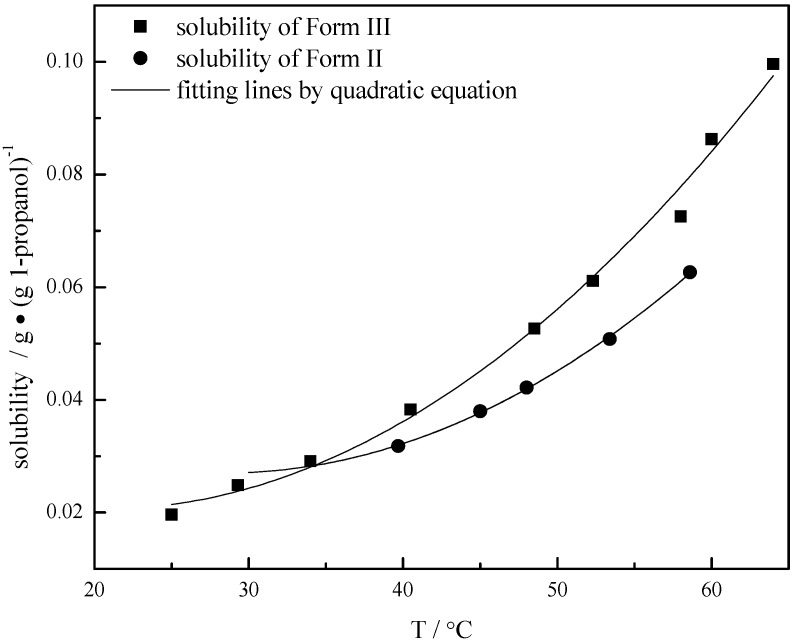
The solubility-temperature diagram and fitting curves for carbamazepine of Forms II and III.

Precisely known amounts of solid CBZ of pure Form II and Form III crystals were added to 1-propanol in the crystallizer and maintained for at least 1 h. Then the temperature of the crystallizer was slowly increased at the rate of 0.05 °C/min until all the excess crystals were dissolved, which was monitored by the FBRM signal change. The temperature at this time was recorded as *T*_s_. The solute concentration at this temperature was calculated by the FTIR model and was considered to be the solubility at *T*_s_, which is shown in Figure 5 and Table 2. Because of transformation problems, the solubility of Form II could not be determined below 40 °C. The thermodynamic relationship between the two polymorphic forms was determined by plotting the solubility data using a quadratic equation. A transition point *T*_t_ between the two polymorphic forms was evaluated at 34.2 °C by extending the quadratic fitting trend line of the Form II solubility. The fact that a transition point exists below the melting point of CBZ (191–192 °C) confirms that the two forms are enantiotropically related. At the transition temperature point the solubilities of the two forms are the same. The solubility of Form III is higher than that of Form II in 1-propanol above the transition temperature, while below the transition temperature, the solubility of Form III is lower than that of Form II.

**Table 2:** The solubility and metastable limit data of carbamazepineForms II and III in 1-propanol.

T/°C	Solubility of Form II/g ∙ (g 1-propanol)^−1^	T/°C	Metastable limit of Form II/g ∙ (g 1-propanol)^−1^	T/°C	Solubility of Form III/g ∙ (g 1-propanol)^−1^	T/°C	Metastable limit of Form III/g ∙ (g 1-propanol)^−1^
39.7	0.0318	39.2	0.04777	25.0	0.0196	22.0	0.03407
45.0	0.03796	45.9	0.06155	29.3	0.02485	27.7	0.03909
48.0	0.04215	52.3	0.07261	34.0	0.02909	34.2	0.04595
53.4	0.05077	58.0	0.08335	40.5	0.03831	38.5	0.04806
58.6	0.06267	--	--	48.5	0.05267	--	--
--	--	--	--	52.3	0.06111	--	--
--	--	--	--	58.0	0.07252	--	--
--	--	--	--	60.0	0.08632	--	--
--	--	--	--	64.0	0.09961	--	--

### 3.3. Metastable Limits of Carbamazepine Polymorphs

**Figure 6:**
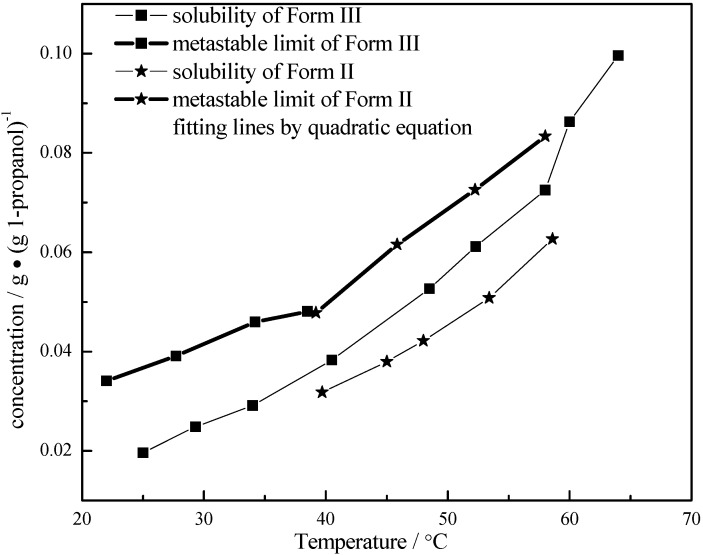
The metastable limits and fitting curves for Carbamazepine of Forms II and III.

Saturated CBZ solutions were prepared and then heated to 3 °C above the saturation temperature and maintained at that temperature for at least 1 h. The solution was then cooled down at the rate of 0.05 °C /min till visible crystals were detected by the FBRM at temperature *T*_l_, and the concentration was determined by FTIR, which was defined as the primary metastable limit at *T*_l_. Figure 6 and Table 3 show the metastable limits. There was a point of intersection at 39.9 °C of the quadratic fitting curves for CBZ metastable limits of Forms II and III. It can be inferred that, Form II will be inclined to nucleate if only primary nucleation happened above the intersection point. On the contrary, Form III tends to nucleate below the intersection point.

**Table 3:** Regression parameters for Raman correlation.

Parameters	*A*	*B*	*R*^2
**values**	9.71 × 10^−6^	7.51 × 10^−4^	0.998
**error**	3.87 × 10^−6^	0.545 × 10^−4^	

### 3.4. Quantitative Analysis of Polymorphic Transformation

**Figure 7:**
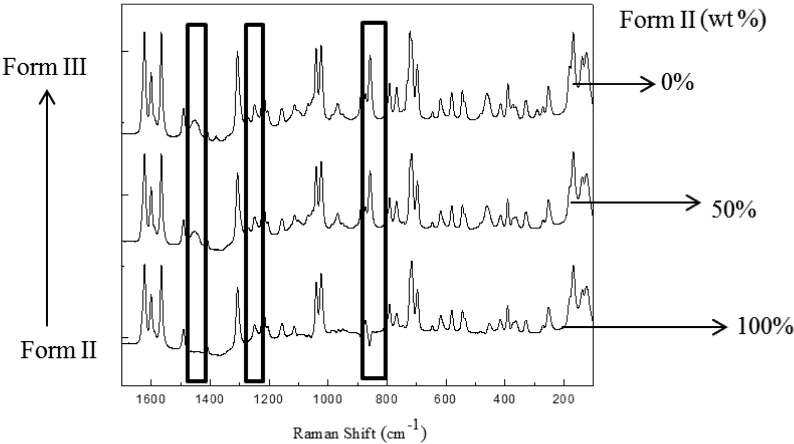
The Raman spectrum of polymorphs at different ratios.

To investigate the potential of the Raman technique for quantitative polymorph analysis, several calibration standards containing 0 to 100 wt% of form II with 10% increments in mixtures of Form II and Form III were prepared. A typical trend in three characteristic peaks of a mixture of Form II and Form III of carbamazepine is shown in Figure 7. Distinctive peaks of the Raman trace at 857 cm^−1^ and 1450 cm^−1^ for the Form III and Form II respectively can be readily observed. These Raman peaks are relatively sensitive to the changes in weight ratios between Form III and Form II. As discussed in the literatures [6,8], the Raman intensities depend not only on the concentration of individual polymorphic forms but also on the overall solid content, particle size of the solid phases and temperature in the mixtures. The intensity at 1271 cm^−1^ was found to be suitable for background to reduce these effects. The following equation and corresponding regression parameters in Table 3 were used to quantify the polymorphs during the polymorphic transformation process. In equation 2, *H* represents the Raman intensity of each peak, *Y* represents the mass fraction of solid Form II in suspension. To reduce the effect of the overall solids concentration and solution content, the results were divided by the calculated value of pure form II in each run.

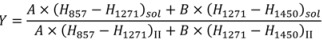
(2)


### 3.5. Real-Time Monitoring of Polymorphic Transformation of Carbamazepine during Seeded Isothermal Crystallization

Three experiments as outlined in Table 4 were performed to examine the polymorphic transformation of carbamazepine during isothermal crystallization (at 25 °C) in the presence of different percentages of Form II seeds.

**Table 4:** Experiment conditions for polymorph transition trials in the isothermal crystallization.

Run Number	Initial solution concentration (g/g 1-propanol)	Supersaturation for Form III (g/g)	Relative seed mass of Form II (g Form II/g solute)
1	0.0275	0.2791	7%
2	0.0275	0.2791	10%
3	0.0275	0.2791	15%

#### 3.5.1. FBRM Results of Polymorphic Transformation in Seeded Isothermal Crystallization

The difference between the solubility of the two forms at 25 °C is relatively large and the transition from the form II to form III should be slow enough to permit accurate measurements of the system behavior. Runs 1-3 were conducted in such a way that the polymorphic transformation was observable after seeding the saturated solution with Form II. For this set of runs, we used the same initial solute concentration, which was at the saturation point with respect to Form II but supersaturated for Form III. There was no driving force in the solution for growing seeds of Form II crystals and Form II showed a tendency to transform to stable polymorphic structure.

**Figure 8:**
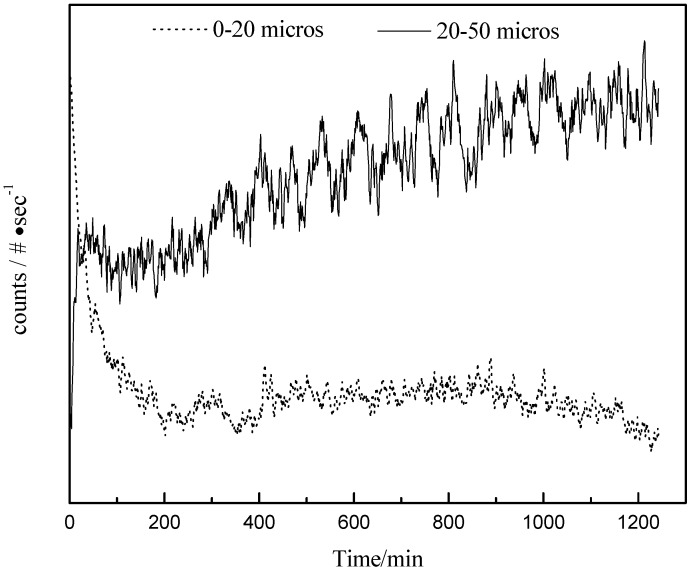
Development of chord length counts with time during Run 2.

In Run 2, on addition of the Form II seed crystals, there was an increase in the particle counts (#/s in 20–50 microns channel) measured by the FBRM, however, the particle counts in the channel of 0–20 microns decreased sharply, as shown in Figure 8. The same trend was observed in Runs 1 and 3. It is speculated that the increase in particle count is due to the nucleation of Form III and the decrease in particle count in the smaller size range is a result of the dissolution of the Form II.

**Figure 9:**
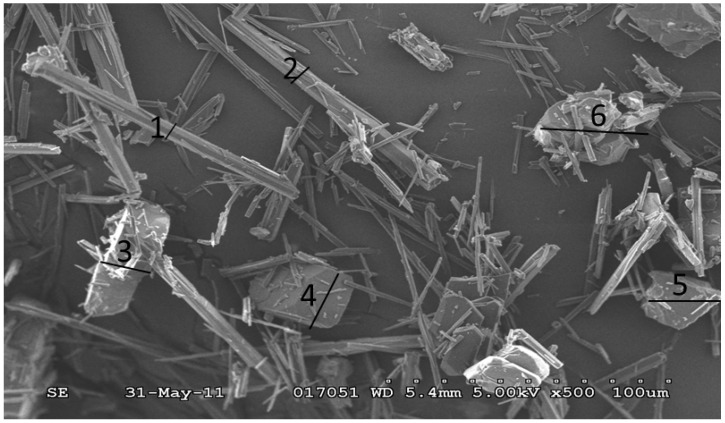
SEM picture of the sample taken at 20 min in Run 2. Crystals 1 and 2 are considered to be Form II and 3-6 as Form III.

Figure 9 shows one of the electron micrographs of the crystals in the suspension at 20 min after seeds were added in Run 2. As can be seen, some plate-like crystals (Form III) appeared among the needlelike crystals (Form II). From image analysis data, the width of particles 1–6 in Figure 9 were about 6.7 μm, 10.7 μm, 16.0 μm, 25.3 μm, 26.7 μm and 35.0 μm respectively . From this morphology, it is speculated that the particles 1–2 were Form II and particles 3–6 were Form III carbamazepine. That is to say, the width of most of the Form II crystals was smaller than 20 µm, while the width and length of most Form III, was larger than 20 µm. When measuring needlelike crystals, FBRM chord length is a function of the width of these needles as well as their length [5]. Hence, crystals in the channel of 20–50 microns increased when Form III nucleated and grew while the particle counts in the channel of 0–20 microns decreased when Form II dissolved.

#### 3.5.2. Raman and FTIR Results of the Polymorph Transformation in Seeded Isothermal Crystallization

**Figure 10:**
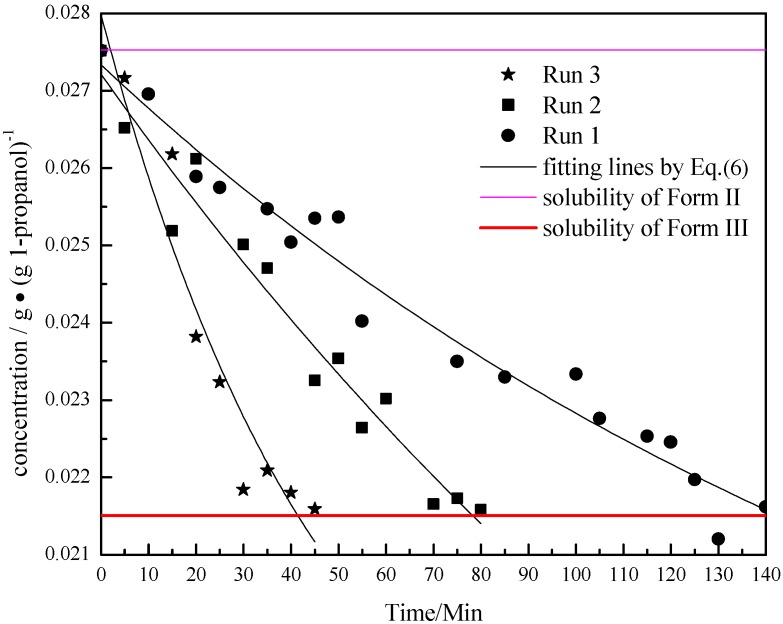
The comparison between the concentration profiles measured by ATR-FTIR during Runs 1, 2 and 3.

**Figure 11:**
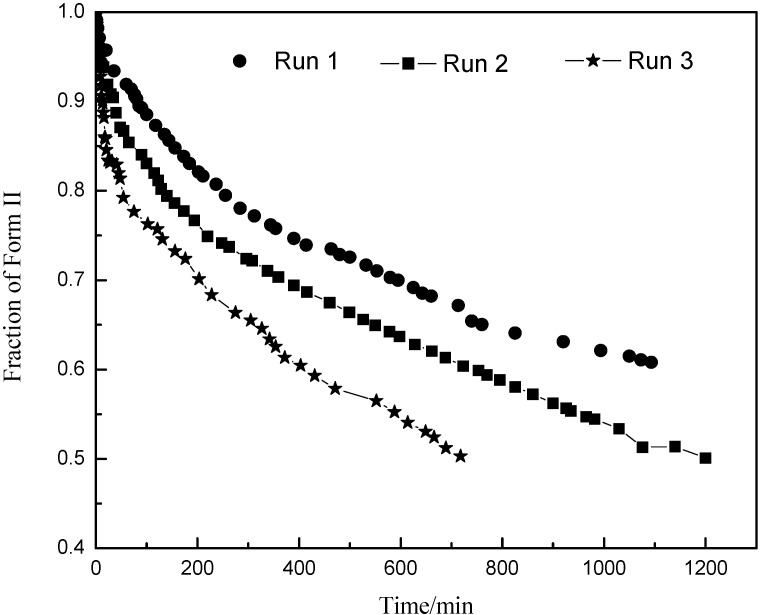
The conversion ratio of the two forms measured by Raman during Runs 1, 2 and 3.

The concentration profile of the solution at the beginning of the polymorph transformation for this set of runs is shown in Figure 10. From the results of FBRM and microscopic pictures, it is speculated that at the beginning, the supersaturation was used for the growth of Form III crystals, while Form II crystals dissolved as the concentration decreased. It can be inferred from Figure 11 that the deposition rate of Form III crystals was faster than the dissolution rate of Form II crystals because the concentration of the solution decreased with time. Furthermore, the speed of consumption of supersaturation was quicker after adding a greater amount of Form II seed. This could be explained by the following very simplified model.

A mass balance for carbamazepine Form II and Form III in the isothermal batch crystallizer could be expressed by Equation (3) as:

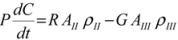
(3)
where A_II_ and A_III_ are the total surface area of Form II and Form III crystals in vessel respectively; *ρ*_II_ and *ρ*_III_ are the crystal densities; *C* represents the concentration of CBZ in solution, g/g 1-propanol; P is the mass of 1-propanol in solution in vessel. The dissolution rate for Form II and precipitation rate for Form III may be described by the following power-law expressions:

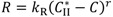
(4)

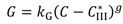
(5)
where 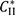 and 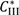 are solubilities of Form II and Form III respectively, g/g 1-propanol; *R* is the dissolution rate for Form II and *G* is the precipitation rate for Form III, both as g m^−2^ s^−1^; *k*_R_ and *k*_G_ are rate coefficients respectively. If it is assumed that A_II_ and A_III_ are constant with time (a sweeping assumption) and both the dissolution and growth processes are first order (*r* = 1; *g* = 1), substituting Equation (4) and Equation (5) into Equation (3) and integrating gives:

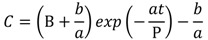
(6)
where 
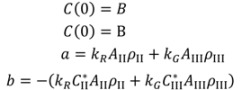


Equation (6) shows that the concentration *C* falls exponentially with time *t*. The curves shown in Figure 10 are also approximately exponential before 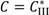. As the amount of seeds is increased the depletion curves (Figure 10) falls faster. The calculated values were also fitted by Equation (6), all the corresponding R^2 were above 0.90. Using the definition above for *a*, the model indicates that as more seed is added, A_II_ and thus *a* would increase and so the concentration would fall faster for higher amounts of seed, as shown in Figure 10. Note that by assuming A_II_ and A_III_ do not very with time, the model is highly approximate. The ratio of the two forms suspended in the solution can also be calculated from Raman spectra and compared with Figure 11. From Figure 8,Figure 9,Figure 10,Figure 11, we can conclude that the nucleation of Form III started immediately after Form II seeds were added in the saturated solution and the dissolution of Form II seeds followed thereafter.

## 4. Conclusions

Two pure forms of CBZ (Forms II and III) were produced and characterized by XRD, DSC and SEM. Two different *in-situ* analytical measurement techniques, ATR-FTIR spectroscopy and FBRM were combined to determine the solution thermodynamics of carbamazepine Form II and Form III. The transition temperature between Form II and Form III was determined at about 34.2 °C showing Form II is the thermodynamic stable form at higher temperatures and providing the possibility of obtaining the metastable Form II of CBZ.

*In-situ* Raman spectroscopy was used successfully in the isothermal crystallization of carbamazepine. The counts measured by FBRM were used together with the off-line microscopic pictures to detect the dissolution of metastable form and nucleation of the stable form in all runs. From the trends of Raman and FTIR in isothermal crystallization, it was noted that the rate of transformation process was affected by the amount of added Form II seeds. Higher Form II seed loading resulted in faster transformation from Form II to Form III.
